# *Anaerobutyricum hallii* promotes the functional depletion of a food carcinogen in diverse healthy fecal microbiota

**DOI:** 10.3389/frmbi.2023.1194516

**Published:** 2023-09-18

**Authors:** Alejandro Ramirez Garcia, Anna Greppi, Florentin Constancias, Hans-Joachim Ruscheweyh, Julie Gasser, Katherine Hurley, Shana J. Sturla, Clarissa Schwab, Christophe Lacroix

**Affiliations:** ^1^ Laboratory of Food Biotechnology, Department of Health Sciences and Technology, ETH Zürich, Zürich, Switzerland; ^2^ Laboratory of Toxicology, Department of Health Sciences and Technology, ETH Zürich, Zürich, Switzerland; ^3^ Institute of Microbiology, Department of Biology, and Swiss Institute of Bioinformatics, ETH Zürich, Zürich, Switzerland; ^4^ Department of Biological and Chemical Engineering, Aarhus University, Aarhus, Denmark

**Keywords:** dietary carcinogens, PHIP, glycerol/diol dehydratase, reuterin, *Anaerobutyricum hallii*

## Abstract

**Introduction:**

*Anaerobutyricum hallii* is a human gut commensal that transforms the heterocyclic amine 2-amino-1-methyl-6-phenylimidazo [4,5-b] pyridine (PhIP), a carcinogen from cooked meat. The transformation mechanism involves the microbial production of acrolein from glycerol, and its conjugation with PhIP, thus blocking its mutagenic potential. A potential cancer prevention strategy could therefore involve supplementing complex human microbial communities with metabolically competent bacteria such as *A. hallii* that can deplete PhIP. However, it has not been established how the proportion of *A. hallii* in diverse healthy human gut microbial communities relates to functional capacity for PhIP transformation and, moreover, how supplementing microbiomes with *A. hallii* affects this function.

**Methods:**

In this study, shotgun metagenomics was used to study taxonomic profiling, the abundance of glycerol/diol dehydratase (*gdh*)-harboring taxa, the proportion of resident *A. hallii*, and the reconstruction of *A. hallii* population genomes in the fecal samples of 20 healthy young adult donors. Furthermore, the influence of supplementing 10^6^ cells/mL of *A. hallii* DSM 3353 with diluted fecal microbiota was characterized.

**Results and discussion:**

Six microbiota were assigned to *Bacteroides*, nine to *Prevotella*, and five to *Ruminococcus* by enterotype-associated clustering. The total number of *gdh* copies in the 20 fecal microbiota expressed per 10^10^ bacterial cells ranged between 1.32 × 10^8^ and 1.15 × 10^9^. Eighteen out of the 20 donors were dominated by *A. hallii*, representing between 33% and 94% of the total *gdh* relative abundance of the samples. The microbiota with low *A. hallii* abundance (i.e., with a relative abundance < 1%) transformed less PhIP than the microbiota with high *A. hallii* abundance (i.e., with a relative abundance > 1%). Furthermore, supplementing the low-*A. hallii*-abundant microbiota with glycerol significantly increased the PhIP transformation capacity after 6 h while reducing total short-chain fatty acid (SCFA) levels, which is most likely due to acrolein production. Although acetate decreased in all microbiota with glycerol and with the combination of glycerol and *A. hallii*, for most of the microbiomes, butyrate production increased over time. Thus, for a significant number of diverse healthy human fecal microbiomes, and especially when they have little of the taxa to start with, supplementing *A. hallii* increases PhIP transformation. These findings suggest the need to test *in vivo* whether supplementing microbiomes with *A. hallii* reduces PhIP exposure.

## 1 Introduction

The human gut microbiota comprise a vast number of microorganisms that metabolize dietary substrates and produce numerous compounds, thus influencing host health (Ursell et al., 2012). Gut bacteria harboring cobalamin-dependent glycerol/diol dehydratases (GDHs) (EC: 4.2.1.28/30) metabolize dietary or endogenous glycerol to 3‐hydroxypropanal (3‐HPA), which can be transformed into 1,3-propanediol (1,3-PD) or can spontaneously degrade to acrolein in physiological conditions (Engels et al., 2016b). Enzymes responsible for glycerol transformation via GDH are encoded by the *pdu-cob-cbi-hem* operon, which contains the genes required for cobalamin biosynthesis (*cob-cbi*) and for GDH (pduCDE) (Morita et al., 2008; Santos et al., 2008; Sriramulu et al., 2008). Acrolein is a highly reactive unsaturated aldehyde that exhibits cytotoxic and broad-spectrum antimicrobial activities (Stevens and Maier, 2008; Engels et al., 2016b; Zhang and Schwab, 2022). In addition, acrolein has recently been reported to be involved in the detoxification of xenobiotic heterocyclic amines (HCAs) (Zhang et al., 2019a; Zhang et al., 2019b), which can be produced during the cooking of meat due to the high-temperature pyrolysis of amino acids and are suggested to be involved in the initiation of colorectal carcinogenesis (CRC) (Bouvard et al., 2015; Dolan et al., 2021; Kang et al., 2022). The transformation of HCA yields acrolein conjugates (Beer et al., 2017) with reduced cytotoxicity and mutagenicity, and may be beneficial for the host by decreasing HCA exposure and thus reducing CRC risk (Zhang et al., 2019b).

*Anaerobutyricum* is a commonly occurring genus within human gut microbiota (Almeida et al., 2021) and yet consists of only two species: *Anaerobutyricum hallii* (formerly *Eubacterium hallii*) and *Anaerobutyricum soehngenii* (Shetty et al., 2018). Both strains (i.e., DSM 33523 and L2-7, also known as DSM 17630) were isolated from human feces and showed GDH activity (Engels et al., 2016b). *A. hallii* is considered a candidate biotherapeutic due to its ability to produce the short-chain fatty acids (SCFAs) butyrate and propionate, which contribute to intestinal homeostasis (Duncan et al., 2004; Muñoz-Tamayo et al., 2011; Scott et al., 2014; Engels et al., 2016b). Moreover, among gut bacteria, *A. hallii* is reported to be one of the most efficient taxa in the transformation of the HCA 2-amino-1-methyl-6-phenylimidazo [4,5-b] pyridine (PhIP) (Fekry et al., 2016). PhIP is produced by Maillard reactions between glucose, phenylalanine, and creatinine, and is the most abundant HCA produced during the cooking of meat and fish (Felton et al., 2000). On the basis of sufficient evidence in experimental animals, PhIP has been classified since 1993 as a possible human carcinogen (IARC Working Group et al., 1993). Since then, there have been numerous mechanistic data supporting its genotoxicity (Masumura et al., 1999; Tanaka et al., 2005; Bellamri et al., 2021). PhIP can be transformed by *A. hallii* into its acrolein conjugate 7-hydroxy-5-methyl-3-phenyl-6,7,8,9-tetrahydropyrido[3′,2′:4,5]imidazo[1,2-a]pyrimidin-5-ium chloride (PhIP-M1) (Fekry et al., 2016; Zhang et al., 2019b). On the basis of metagenomic predictions, we previously suggested *A. hallii* to be a key microbe in GDH-mediated glycerol metabolism in the gut, followed by *Clostridiales* spp. and *Blautia* spp., and that GDH activity could be enhanced *in vitro* by supplementing three adult fecal microbiota with *A. hallii* in batch fermentation (Ramirez Garcia et al., 2021).

In this study, we tested the hypothesis that PhIP transformation by human fecal microbiota correlates with the *A. hallii* proportion and supplementing various complex microbiomes with *A. hallii* can increase their capacity to transform PhIP. Thus, we used shotgun metagenomics to quantify resident *A. hallii* abundance and the diversity of *A. hallii* populations, and analyzed the *gdh* community in a broader panel composed of 20 diverse fecal microbiota from healthy adults. The fecal microbiota composition of 20 adult donors was characterized, and the microbiota were divided into two groups, high and low, based on the proportion of resident *A. hallii*. Next, we conducted batch fermentations of all 20 fecal microbiota in the presence of PhIP and glycerol and with or without *A. hallii* supplementation. The PhIP transformation capacity, glycerol metabolites, and SCFAs produced during batch fermentations were evaluated based on these two groups and related to the *A. hallii* resident population or supplementation.

## 2 Materials and methods

### 2.1 Fecal sample collection

The fecal samples were collected from 20 healthy adult individuals (median age 30 years) who had not received antibiotics in the previous 3 months. Informed written consent was obtained from each donor, and a donor number was randomly assigned for blinding. Fecal samples were collected by donors immediately after defecation using a sterile container. An atmosphere generator (AnaeroGen™; Thermo Fisher Scientific, Pratteln, Switzerland) was added into the container to produce anaerobic conditions during transportation. The samples were transferred within 2 hours into an anaerobic chamber (10% CO_2_, 5% H_2_, and 85% N_2_) (Coy Laboratories. Grass Lake, MI, USA), aliquots were stored at −80°C for DNA isolation, and the samples were used to immediately inoculate batch fermentations, as outlined below. The Ethics Committee of ETH Zürich exempted this study from review because the sample collection procedure was not performed under conditions of intervention and was done anonymously.

### 2.2 Fecal batch fermentation

A 10% w/v (weight/volume) fecal suspension was prepared inside an anaerobic chamber by resuspending 1 g of fresh feces in 10 mL of sterile peptone water in a 50 mL sterile Falcon™ tube with four sterile glass beads (5 mm). The tube was vortexed for 5 min and the mixture was left to stand for 5 min to allow the heavy particles to settle. The resulting supernatant was used to inoculate (0.1% w/v) serum flasks containing 20 mL of Macfarlane medium prepared according to Bircher et al. (2018b) and supplemented with 200 nM of PhIP (Chemie Brunschwig AG, Basel, Switzerland) and 100 mM of glycerol (Sigma-Aldrich, Buchs, Switzerland). This concentration of glycerol was chosen based on previous reports (from De Weirdt et al., 2010; Fekry et al., 2016; and Engels et al., 2016a), and was the minimum dose at which PhIP transformation was detected. The fermentations in the Macfarlane medium without added glycerol served as a control. The additional fermentations were supplemented with 1% of a 16-h overnight culture of *A. hallii* DSM 3353 [final concentration in the medium measured by quantitative PCR (qPCR) as 10^6^ cells/mL] grown in a yeast extract—casein hydrolysate—fatty acid (YCFA) medium prepared as previously described (Bircher et al., 2018a). Triplicate fermentations were incubated at 37°C with magnetic stirring (120 rpm) and sampled and analyzed as previously described (Ramirez Garcia et al., 2021). Briefly, 1-mL samples were taken after 0 h, 6 h, and 24 h of fermentation using sterile syringes flushed with CO_2_ and centrifuged in sterile Eppendorf™ Tubes™ (14,000 × g for 10 min at 4°C).

### 2.3 DNA isolation, quantitative PCR, and shotgun metagenome sequencing

DNA was isolated from the pure culture of *A. hallii* inoculum, from the fecal samples, and from the fermentation samples using the FastDNA™ SPIN Kit for Soil (MP Biomedicals, Zurich, Switzerland), following the manufacturer’s protocol. The concentration of the isolated fecal DNA was assessed with a NanoDrop^®^ 1000 instrument and a Qubit™ 4 fluorometer (Thermo Fisher Scientific), whereas the DNA integrity was verified via gel electrophoresis. A previously established qPCR assay was used to quantify the abundance of *A. hallii* in the overnight culture used for *A. hallii* supplementation, using specific primers for *gdh* (forward primer 5′-CGTTATGCTCCATTTAATGCT-3′ and reverse primer 5′-CCAAGGAGTATCATCACCATC-3′) (Ramirez Garcia et al., 2021). The reactions were performed using a Roche LightCycler^®^ II device (Roche Diagnostics, Rotkreuz, Switzerland). For shotgun metagenome sequencing, all fecal samples were diluted to 25 ng/µL^−1^ and sent for sequencing (Novogene Co. Ltd., Cambridge, UK), with the number of reads/samples ranging from 405,268,068 to 620,549,190. The sequencing was performed using the Illumina NovaSeq 6000 platform in 150-bp paired-end mode. The number of generated raw read pairs ranged between 405 million and 620 million, with an average of 466 million.

### 2.4 Gene catalog annotation and quantification

The quality control of raw reads was performed with BBMap (v.38.71) by removing adapters, reads mapping to the human genome, and low-quality reads (Bushnell et al., 2017). The filtered reads were merged with a minimum overlap of 16 bases and were assembled using the SPAdes assembler (v3.14) in metagenomic mode (Bankevich et al., 2012). Gene calling on the assembled scaffolds was performed using Prodigal (Hyatt et al., 2010), and the genes were clustered using CD-HIT-EST (Li and Godzik, 2006) with a cutoff value of 95% identity and 90% overlap of the shorter gene. The reads from all samples were mapped against all representative genes. To account for the differences in gene size, the number of inserts mapped were normalized to represent 1,000-bp genes. The abundance of each gene in each sample was normalized using the median abundance of the 10 single-copy marker genes described by Milanese et al. (2019) to estimate gene copies per bacterial cells, which was expressed per 10^10^ cells in the manuscript.

### 2.5 Metagenome-based taxonomic profiling and identification of *gdh*-harboring taxa

The taxonomic profiles for each sample were generated by profiling quality-controlled metagenomic reads using the metagenomic-based operational taxonomic units (mOTUs) profiler version 3.0.1 using default parameters (Milanese et al., 2019).

The contigs harboring genes belonging to the *pduC*/*gdh*-annotated clusters from the gene catalog were identified. The collections of metagenome-assembled genomes (MAGs) that were specific for each donor (i.e., list of contigs belonging to a particular MAG) were then used to link *pduC/gdh* genes to bacterial populations and their Genome Taxonomy Database (GDTB) assignments. When the identified contigs were not assigned to any MAG, the taxonomy of the gene belonging to *pduC*-annotated clusters was determined by aligning the corresponding contig sequence against the National Center for Biotechnology Information (NCBI) non-redundant nucleotide database (nt).

### 2.6 Microbiota community analysis

The merged mOTU profiles were loaded into R (The R Foundation for Statistical Computing, Vienna, Austria) as phyloseq objects (McMurdie and Holmes, 2013), and data analysis was conducted using DivComAnalyses R functions (Constancias and Sneha-Sundar, 2022). The taxonomic structures were clustered to determine the enterotype, as previously defined (Arumugam et al., 2011). The principal coordinate analysis was used to depict similarity between donors’ microbiota. The correlation between family-level proportion and ordination pattern was evaluated, and the top 10 significantly correlated families were displayed (*p* < 0.05).

The association between microbiota and metadata (i.e., PhIP transformation, age, diet) was investigated at the alpha/beta and mOTU abundance levels. A permutational multivariate analysis was used to identify marginal associations between community structure and covariates. In a second step, distance-based partial redundancy analysis was used to evaluate the association between microbiota structure and PhIP transformation while controlling for differences in the microbiota associated with enterotypes.

### 2.7 Metagenome-assembled genome binning, quality filtering, and annotation

The assembled scaffolds were length filtered (≥ 1,000 bp), and quality-controlled metagenomic reads from all samples were individually mapped against the scaffolds of each sample using the Burrows–Wheeler aligner (v0.7.17-r1188) (Li and Durbin, 2009), allowing for secondary alignments. The alignments were filtered to > 45 bases, with an identity of ≥ 97% and covering ≥ 80% of the read sequence. To provide within- and between-sample coverages for each scaffold, the resulting binary alignment map (BAM) files from the previous step were processed using MetaBAT2 (v2.12.1) (Kang et al., 2019). The scaffolds on all samples were finally binned with MetaBAT, and the quality of the resulting bins was assessed using CheckM (v1.0.13) (Parks et al., 2015). Only the bins with a completeness level of ≥ 50% and a contamination level of < 10%, and which belonged to the domains of Bacteria or Archaea as reported by Anvi’o (v5.5.0) (Eren et al., 2015) were used as MAGs for further analysis. The taxonomic annotation of MAGs was performed using GTDB-Tk (v1.5.0, database R06-RS202) (Chaumeil et al., 2019). The MAGs assigned to the *Anaerobutyricum* genus were subjected to the Anvi’o pangenomic workflow, which identified two fragmentated MAGs in donors 2 and 15. The FASTA files corresponding to these MAGs were concatenated and subjected to CheckM and GToTree together with the other MAGs for final completeness and contamination estimation.

To identify the presence of the *pdu-cob-cbi-hem* operon in the MAGs assigned to the genus *Anaerobutyricum*, the MAGs were annotated using Prokka (Seemann, 2014) and the resulting annotation was manually screened for the *pdu-cob-cbi-hem* operon genes. Publicly available genomes of the strains *A. hallii* DSM 3353 and *A. soehngenii* L2-7 (DSM 17630) were annotated and used as reference strains.

### 2.8 Determination of glycerol consumption and metabolite production

The consumption of glycerol and the production of SCFA and 1,3-PD was monitored using high-pressure liquid chromatography—refractive index (HPLC-RI), as described before (Zhang et al., 2019b). In brief, culture supernatant was filtered with a 0.45-µm nylon filter (Phenomenex, Basel, Switzerland), diluted 1: 1 in H_2_O, and transferred into an HPLC vial with a 250-µL conical glass insert (BGB Analytik, Boeckten, Switzerland). The samples were eluted isocratically with 10 mM H_2_SO_4_ at 40°C and at a flow rate of 0.4 mL/min^−1^ using an Aminex HPX-87H column (300 mm × 7.8 mm, with a 9-µm particle size; Bio-Rad Laboratories, Reinach, Switzerland). Glycerol, 1,3-PD, and SCFA were quantified by independent calibration with external standards (all obtained from Sigma-Aldrich). The detection limits were 0.7 mM for acetate, propionate, and butyrate, 0.9 mM for glycerol, and 0.2 mM for 1,3-PD.

### 2.9 Quantification of PhIP transformation by nanospray liquid chromatography—tandem mass spectrometry

The transformation of PhIP into PhIP-M1 was determined via nanospray liquid chromatography coupled to tandem mass spectrometry (nanoLC-MS)/MS using a nanoACQUITY Ultra Performance LC™ (UPLC^®^) System (Waters Corporation, Milford, MA, USA) attached to a TSQ Vantage™ triple-stage quadrupole mass spectrometer (Thermo Fisher Scientific), as described previously (Ramirez Garcia et al., 2021).

The calibration curves were generated using the ratio between the peak area of 200 nM of the internal standard 5-amino-6-methylbenzamidazolone (AMBI) and of 5–250 nM PhIP and PhIP-M1. The transformation of PhIP was reported as a percentage of the initial PhIP transformed to PhIP-M1.

### 2.10 Statistical analyses

R (version 4.2.2) was used for the statistical analyses. Based on the Shapiro–Wilk test, the data displayed non-normal distribution. Therefore, the differences between the groups for PhIP transformation, glycerol consumption, SCFAs, 1,3-PD, and *A. hallii* abundance (low vs. high) were assessed using a two-sided non-parametric Wilcoxon test. Adjusted *p-*values of ≤ 0.05 were considered to be significant. Spearman-ranked correlations were used to investigate associations between (i) observed species (i.e., number of mOTUs) and Shannon and Simpson diversity indices (i.e., diversity of mOTUs in a community), or (ii) mOTU proportions of the top 200 mOTUs and PhIP transformation for which only correlations with adjusted *p*-values of ≤ 0.05 and |Spearman’s rho| ≥ 0.6 were considered. Computed *p*-values were false discovery rate (FDR)-adjusted for multiple comparisons.

### 2.11 Sequence deposits

Raw sequence data were deposited with NCBI (in the Sequence Read Archive) and are available under BioProject PRJNA720271.

## 3 Results

### 3.1 Microbial community structure of fecal microbiota

**Figure 1:**
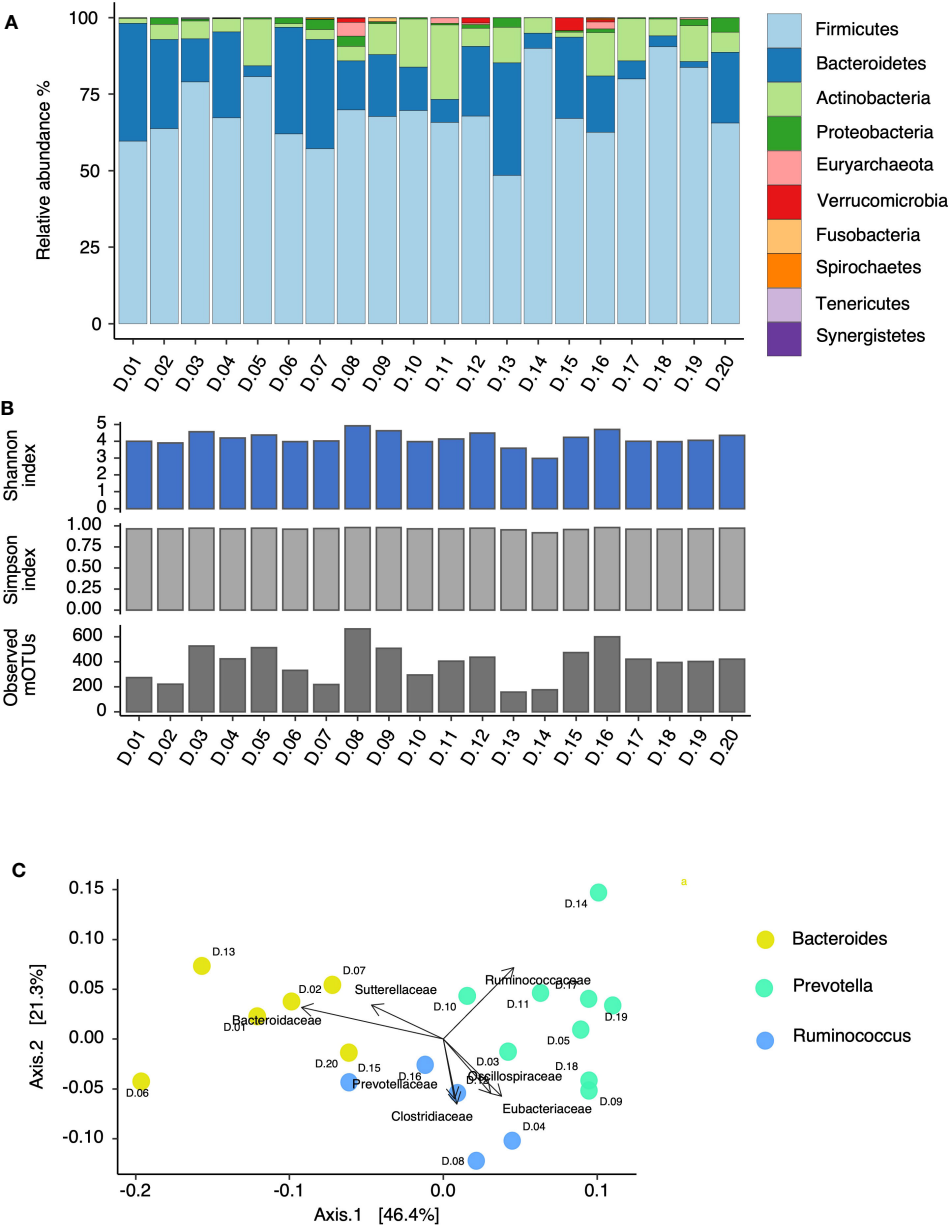
Microbial community structure of 20 human fecal donors (D) analyzed by shotgun metagenomics. Relative abundance at the phylum level **(A)**. Shannon index, Simpson index, and the number of observed species **(B)**. Principal coordinate analysis displaying enterotype clustering based on Jensen–Shannon divergence computed at the genus level, and the top seven significantly correlated families (adjusted *p*-value ≤ 0.05) **(C)**.

To understand the diversity of the microbiomes used for this study, the microbial composition of fecal samples collected from 20 young healthy adults was assessed by shotgun metagenomics (**Figure 1**). Collectively, we identified 1,839 individual mOTUs within all 20 fecal samples. In the samples, taxa belonging to the Firmicutes phylum were the most abundant of all microbiota, with relative abundances of between 48.4% and 90.5%. This was followed by taxa belonging to the Bacteroidetes phylum, which ranged between 1.9% and 38.4% (**Figure 1A**). With regard to community diversity values, these ranged from 2.9 to 4.9 by the Shannon index, and 0.91 to 0.98 by the Simpson index, and individual fecal microbiotas harbored from 162 (D.13) to 701 (D.08) mOTUs (**Figure 1B**). To identify enterotype-associated clusters of the microbiota, a beta-diversity analysis (i.e., comparison of samples based on overall microbiota similarity) based on the Jensen–Shannon divergence was computed at the genus level. The analysis assigned six microbiota to the *Bacteroides* enterotype, nine to *Prevotella*, and five to *Ruminococcus* (**Figure 1C**).

### 3.2 Proportion and distribution of *gdh* in fecal microbial communities

**Figure 2:**
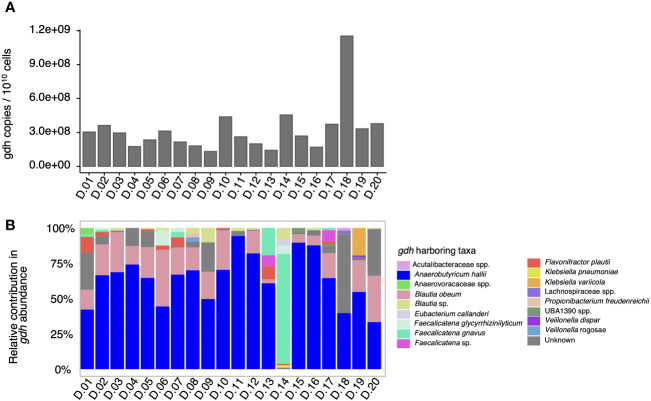
Total *gdh* abundance and *gdh*-contributing taxa quantified by shotgun metagenome sequencing in 20 adult human fecal microbiota donors (D). The total number of *gdh* copies per 10^10^ bacterial cells in the microbiota of each of the 20 donors, which was quantified using the gene catalog approach **(A)**. The relative contribution of *gdh*-harboring taxa based on the MAG catalog and contig-level taxonomy **(B)**.

As a next step, the proportion of taxa contributing to the *gdh* prevalence in each fecal microbiota was evaluated by creating a gene catalog from the shotgun metagenomes to quantify the proportion and distribution of *gdh*-harboring taxa in the fecal microbiota. In the 20 fecal microbiota, the total number of *gdh* copies expressed per 10^10^ bacterial cells ranged from 1.32 × 10^8^ to 1.15 × 10^9^ (**Figure 2A**). When looking into specific *gdh*-harboring taxa, a total of 17 taxa harboring *gdh* [i.e., the propanediol dehydratase large subunit (pduC)—functional annotation from the Kyoto Encyclopedia of Genes and Genomes (KEGG) database K01699] were identified (**Figure 2B**). Specifically, the dominant *gdh*-harboring taxa in the microbiota of 18 donors was *A. hallii*, representing between 33% (D.20) and 94% (D.11) of the total *gdh* relative abundance of the samples. When the most abundant *gdh*-harboring taxon was not *A. hallii*, it was *Faecalicatena gnavus* (78%) for D.14 or a taxon that could not be assigned in D.18, accounting for 56% of the total *gdh* community (**Figure 2B**). To further explore such unassigned *gdh*-harboring taxa in D.18, it was BLASTed and could be identified with *Alkalimarinus* sp. SCSIO (84% identity and 13% electable coverage). Besides *A. hallii* and *F. gnavus*, other taxa that contributed significantly to the *gdh* communities in the fecal microbiomes were *Blautia obeum* (present in the feces of 18 donors, representing between 3% and 40% of the total *gdh*), an unidentified species of the *Blautia* genus (present in the feces of nine donors at between 0.6% and 9% of the total *gdh*), and *Flavonifractor plautii* (present in the feces of eight donors at between 1% and 11% of the total *gdh*) (**Figure 2B**).

### 3.3 Proportion of *A. hallii* population in fecal metagenomes

**Figure 3:**
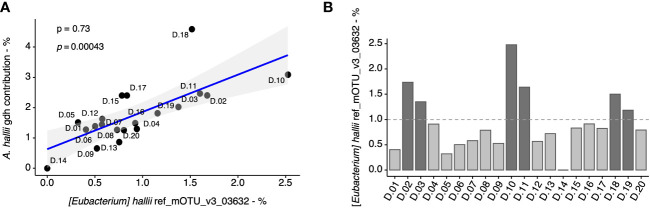
Quantification of *A. hallii* in fecal metagenomes of 20 adult donors. Correlation (Spearman) between the percentage of *gdh* assigned to *A. hallii* by metagenomics based on the gene catalog data, and the percentage of *A. hallii* mOTUs detected by metagenomics using mOTU-based taxonomic profiling in each donors’ sample **(A)**. Spearman’s rho (ρ) and associated *p*-values are displayed. The relative abundance of *A. hallii* [(*Eubacterium*) *hallii* ref_mOTU_v3_03632] in the fecal metagenomes **(B)**.

Given that *A. hallii* was the most abundant *gdh*-harboring taxa in the fecal microbiome, we further estimated its proportion on the basis of both a taxonomic marker (i.e., 16S rRNA) and the proportion of a functional gene of *A. hallii* (i.e., *gdh*) in the fecal samples. At first, we evaluated the coherence of these two approaches and found a significant correlation (*p* = 0.00043), thus validating the results previously obtained for *A. hallii* and *gdh* for all the fecal samples (**Figure 3A**). Based on the *A. hallii* proportion, 1% was arbitrarily chosen to stratify the fecal microbiota in two groups defined as “low” (*A. hallii* < 1%) and “high” (*A. hallii* > 1%), and 14 microbiota were assigned to the “low” group and six microbiota to the “high” group (**Figure 3B**). Importantly, *A. hallii* identified by (*Eubacterium hallii*) mOTU_v3_03632 was the only taxa significantly associated with PhIP transformation in the 20 fecal metagenomes (**Supplementary Table 1**).

### 3.4 Presence of *A. hallii* populations and the *pdu-cob-cbi-hem* operon in fecal metagenomes

To further investigate *A. hallii* populations, we used genome-resolved MAGs to compare the composition of the *cbi-cob-hem-pdu* operon in each fecal sample (**Figure S1**). Overall, binning of the obtained assembled contigs from all samples resulted in 2,891 MAGs. These MAGs were clustered at 95% similarity for species identification, resulting in a collection of 850 species-level MAGs. In all microbiomes, we recovered one MAG belonging to the *A. hallii* species-level MAG cluster. The taxonomy of those MAGs matched with *A. hallii*, with the only exceptions being D.14 and D.03, in which one MAG assigned to an unidentified species of the genus *Anaerobutyricum* was recovered (**Figure S1**). Despite the methodological limitations of MAG reconstruction related to fragmented MAGs, we identified donor-specific *A. hallii* populations that differed in the content of the genes required for acrolein production. Specifically, nine *A. hallii* MAGs harbored a *pdu-cob-cbi-hem* operon with highly similar gene content and organization to *A. hallii* DSM 3553 and *A. soehngenii* L2-7. The *cbi* and *hem* genes were not recovered in MAGs from D.17, D.20, D.10, D.06, or D.19, whereas the same MAGs harbored only two *cob* genes (D.19 only one). (**Figure S1**).

### 3.4 PhIP transformation by human microbiota with glycerol and *A. hallii*

**Figure 4:**
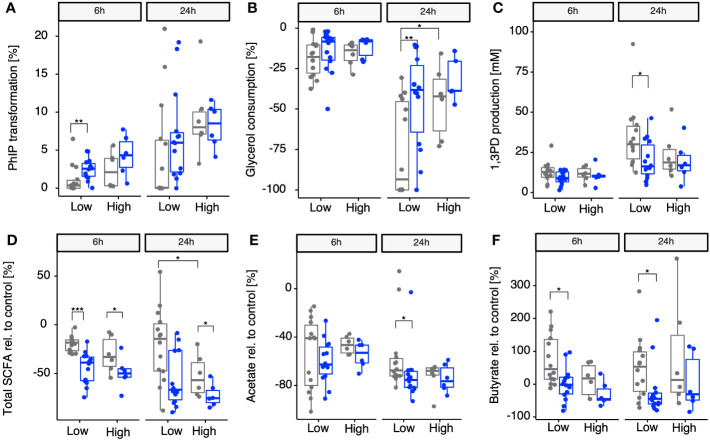
Impact of supplementing microbiomes with *A. hallii* on PhIP transformation, glycerol metabolism, and SCFAs in batch-fermented fecal microbiota. Gray points, no *A. hallii* supplement added. Blue points, *A. hallii* supplement added. Transformation of PhIP (%) by the low (< 1% abundance) and high (> 1% abundance) *A. hallii* abundance group **(A)**, consumption of glycerol **(B)**, and the production of 1,3-PD **(C)** and total SCFAs relative to the control condition, which was the condition in which no glycerol was added to the fermentation **(D)**, acetate relative to the control **(E)**, and butyrate relative to the control **(F)**. Each box represents the median of all donors present in the low or high group. Non-parametric Wilcoxon tests were used to compare each group between *A*. *hallii*-supplemented and non-supplemented microbiota, as well as between non-supplemented microbiota in each *A. hallii* abundance group. Only significant comparisons are represented with adjusted *p*-values ≤ 0.05 (*), ≤ 0.01 (**), and ≤ 0.001 (***).

Once we identified *A. hallii* as being the main contributor of *gdh* activity for 18 fecal microbiota and confirmed that the 20 diverse fecal microbiota of healthy adults could be stratified by proportion of *A. hallii* mOTUs, we hypothesized that the groups would differ in their actual capacity to transform PhIP to PhIP-M1. Thus, fecal batch fermentations were conducted anaerobically in the presence of PhIP (200 mM) and glycerol (100 mM), with or without supplementing additional *A. hallii*, and samples were analyzed after 24 h and 48 h for PhIP transformation (**Figure 4A**). In the presence of glycerol, the microbiota with high *A. hallii* transformed more of the PhIP than the microbiota with low *A. hallii* did [after 6 h, median of 2% versus 0% (*p* = 0.17), and after 24 h, 9% versus 4% (*p* = 0.0508), respectively]. In the same low *A. hallii* group, when fecal microbiota were supplemented with *A. hallii* at a final concentration of 10^6^ cells per mL a significant increase in PhIP transformation was observed after 6 h compared with glycerol alone [median of 4% compared with 0% transformation in the presence of glycerol only (*p* = 0.007)] (**Figure 4A**). Finally, when fecal microbiota were incubated for 24 h in controlled batch fermentations without glycerol, no PhIP transformation was detected (data not shown). In line with the batch fermentation results, PhIP was better depleted with increasing proportions of resident *A. hallii* in all 20 fecal microbiota, at both 6 h and 24 h (**Figure S2A**). This association was not observed for any other taxa. On the other hand, PhIP transformation could not be associated with either alpha- or beta-diversity metrics (**Figure S3** and **Table S1**). Furthermore, no significant correlation between PhIP transformation and community structure was detected when we controlled for variations associated with enterotype (*p* = 0.45; **Table S2**).

### 3.5 Impact of *A. hallii* on microbial glycerol metabolism

For PhIP transformation to occur, acrolein has to be produced from glycerol via the production of the intermediate 3-HPA, leading to the generation of 1,3-PD as the final metabolic product (Vollenweider and Lacroix, 2004). Therefore, besides PhIP transformation, the impact of *A. hallii* on the consumption of glycerol and the production of 1,3-PD was also assessed in the same batch fermentations after 6 h and 24 h. 1,3-PD production was used as a validation for the production of HPA in equilibrium with acrolein, both not detectable in complex cultures (Ramirez Garcia et al., 2021). When stratifying the microbiota in low and high resident *A. hallii*, consumption of added glycerol after 6 h was comparable for both groups. However, aligning with the previous observations, after 24 h, an increase in glycerol consumption was observed in both groups when the microbiota were supplemented with glycerol alone (median of 18% and 14% (*p* = 0.0495) in the low and high groups, respectively) (**Figure 4B**). On supplementing microbiomes with *A. hallii*, a significantly reduced glycerol consumption was observed in only the low *A. hallii* group compared with glycerol alone after 24 h (median of 8% consumed versus 18% consumed in the presence of glycerol alone, **Figure 4B**). In line with this, glycerol consumption significantly positively correlated with resident *A. hallii* proportion after 24 h (*p* = 0.59 and *p* = 0.006; **Figure S2B**). Concurrently, a decrease in 1,3PD production was observed (**Figure 4C**), which negatively correlated with the abundance of resident *A. hallii* in the microbiota (**Figure S2C**).

### 3.6 Impact of glycerol and *A. hallii* on SCFA production

To assess whether the abundance of resident *A. hallii* and supplementing with *A. hallii* played roles in the fermentation activity in the presence of glycerol, total SCFAs (as sums of acetate, propionate, and butyrate) and individual metabolites produced during the fecal fermentations were compared with control fermentations without glycerol. After 6 h, a significant decrease in total SCFAs relative to the control group was observed in both the low (median of −18% versus −39% without and with *A. hallii*, respectively) and high (median of −33% and −50% without and with *A. hallii*, respectively) groups when *A. hallii* was supplemented, as compared with glycerol alone (**Figure 4D**). The same trend was observed after 24 h, when a significant decrease of total SCFA production relative to the control was observed in the presence of glycerol and *A. hallii* in the high group (median of −75%), as compared with glycerol alone (median of −57%). Specifically, acetate production decreased in glycerol-supplemented microbiota of both low- and high-resident *A. hallii* groups after 6 h (median −40% and −46%, respectively) and 24 h (median of −67% and −65%, respectively), as compared with the control. However, when *A. hallii* was further supplemented, a significantly higher decrease in acetate was observed in the low group after 24 h (median of −75% versus 65%) as compared with glycerol alone (**Figure 4E**). Butyrate production increased in both the low and the high groups when glycerol was supplemented after both 6 h (+45% and +16% in the low and high groups, respectively) and 24 h (+53% and +12%, respectively), as compared with the control. However, on supplementing microbiomes with *A. hallii*, butyrate production relative to the control significantly decreased, as compared with glycerol alone, in the low group after 6 h (–2%) and 24 h (–45%) (**Figure 4F**). In alignment with previous observations, a negative correlation was observed between the proportion of resident *A. hallii* and total SCFA production relative to the control after 6 h and 24 h when glycerol was supplemented in the 20 fecal microbiota and the trend was confirmed when *A. hallii* was additionally supplemented after 24 h (**Figure S2D**).

## 4 Discussion

Intestinal microbiota have the potential to alter the harmful effects of food xenobiotics, such as HCA (e.g., PhIP). In this study, we characterized the influence of *A. hallii* on PhIP transformation efficiency and we demonstrated the key role of this taxon in the transformation capacity of 20 diverse human fecal microbiomes. By using shotgun metagenome sequencing, we identified a total of 1,839 species in 20 fecal microbial communities, 17 of which harbored *gdh*. Nevertheless, all tested microbiota produced 1,3PD in the presence of glycerol, the end metabolite of GDH glycerol metabolism. This confirmed previous findings (Zhang et al., 2019b; Ramirez Garcia et al., 2021) indicating that, even though a small number of taxa harbor *gdh*, GDH activity is still a common trait in microbiomes.

Among the *gdh-*harboring taxa identified in the fecal samples, and despite the differences in the fecal microbial composition observed, *A. hallii* was the most abundant in 95% of the samples. The MAG reconstructions further confirmed the persistence of *A. hallii* in all fecal microbiota, with differences in the content and organization of the *pdu-cob-cbi-hem* operon, possibly impacting microbial acrolein production. However, it is worth mentioning that the absence of a gene in the reconstructed MAGs does not necessarily mean that the gene is lacking in the genome of the populations because MAGs are assembled from short-read metagenomic reads, and technical aspects impact MAG binning (Chen et al., 2020).

We observed that PhIP transformation by fecal microbiota, in the presence of glycerol, was significantly correlated only with the proportion of resident *A. hallii* mOTUs and with none of the other 1,838 mOTUs identified. This association may be explained by the high capacity of *A. hallii* to produce acrolein in pure culture (Zhang et al., 2019b). Acrolein is a wide-spectrum antimicrobial, which is active against intestinal bacteria (Cleusix et al., 2007). It may provide a competitive advantage for the growth of *A. hallii* compared with other commensals in the gut when glycerol is present, including those that can metabolize glycerol via glycerol kinase and glycerol dehydrogenase.

Besides transforming PhIP, acrolein accumulation reduced the fermentation activity of all fecal microbiota with a stronger effect and higher acrolein accumulation being observed when the culture was supplemented with *A. hallii*. Likewise, for most microbiota, acrolein accumulation, detected indirectly by PhIP transformation, derived from glycerol metabolism resulted in a significant decrease of acetate and an increase in butyrate. This observation is consistent with the previous observations that supplementing glycerol to microbial communities leads to an increase in butyrate in batch fermentations (De Weirdt et al., 2010; Huber et al., 2020; Ramirez Garcia et al., 2021).

Previous *in silico* data showed decreased *A. hallii* abundance in fecal metagenomes from CRC patients, compared with healthy individuals (Zhang et al., 2019b; Ramirez Garcia et al., 2021). Therefore, *A. hallii* abundance might be a useful marker to estimate intestinal PhIP exposure and risk of CRC initiation. Supplementing fecal samples with *A. hallii*, together with glycerol, led to an overall increase in PhIP transformation, especially in the microbiota harboring an original resident *A. hallii* abundance of lower than 1%. This confirmed the possibility of increasing the GDH activity of complex microbiota by supplementing microbiomes with *A. hallii*, especially in microbiota with low resident *A. hallii*, as we previously suggested, with a limited number of three human fecal microbiota *in vitro* (Engels et al., 2016a; Ramirez Garcia et al., 2021). Overall, 60% of healthy fecal microbiota were capable of transforming PhIP by between 3% and 21% after 24 h in fecal batch fermentations, and 95% transformed PhIP when supplemented with *A. hallii*, indicating that acrolein was produced. However, the measured PhIP transformation and glycerol consumption by the microbiota especially after 24 h, suggested a remarkable donor-dependent response. Taken together, these observations suggest that *A. hallii* persistence in intestinal microbiota is linked to acrolein production and thus on PhIP to PhIP-M1 transformation by the microbiota. However, due to the very high reactivity of acrolein in the tested conditions, we could not quantify the actual production but only estimate it based on the production of 1,3-PD as a final metabolic product and indirectly prove PhIP transformation, as previously demonstrated (Engels et al., 2016b; Ramirez Garcia et al., 2021).

Besides transforming PhIP, acrolein accumulation detected indirectly by PhIP transformation reduced the fermentation activity of all fecal microbiota, with a stronger effect observed when *A. hallii* was further supplemented, increasing acrolein accumulation. Likewise, for most microbiota, acrolein accumulation derived from glycerol metabolism resulted in a significant decrease of acetate and to an increase in butyrate. This observation is consistent with the previous observations that supplementing glycerol to microbial communities leads to an increase in butyrate in batch fermentations (De Weirdt et al., 2010; Huber et al., 2020; Ramirez Garcia et al., 2021).

Despite the potentially beneficial effects of microbial metabolism that promotes PhIP consumption, generation of acrolein should be considered, as it can give rise to cytotoxicity and mutagenicity due to its high electrophilicity (Zhang et al., 2018; Zhang and Schwab, 2022). Its accumulation may damage the intestinal cells by degrading functional proteins (Stevens and Maier, 2008) or increasing intestinal barrier permeability (Chen et al., 2017). In the presence of high glycerol concentrations (up to 600 mM) and of specific single-producer strains, acrolein–DNA adducts can also be formed (Ramirez Garcia et al., 2022). However, such glycerol concentrations, as well as the 100 mM glycerol tested here, are unlikely to be physiologically available to gut bacteria. Furthermore, the added concentration of *A. hallii* (at a final concentration of 10^6^ cells/mL) was high and in the same range as the inoculated fecal microbiota (10^−4^ dilution). It is still unclear what impact the concentration of acrolein, produced in the gut, has on the gut microbiota while keeping the HCA effects intact.

In conclusion, by assessing the transformation of PhIP, a carcinogen in cooked meat, of human fecal microbiota from 20 healthy individuals, we found that the proportion of *A. hallii* in the complex microbiota positively correlates with PhIP transformation. Furthermore, supplementing fecal microbiota with *A. hallii* increased PhIP transformation, indirectly indicating an accumulation of acrolein. Such accumulation resulted in altered microbial metabolic activity. Overall, the results provide a potential target for microbial manipulation by supplementing with *A. hallii*, which might decrease the host exposure to PhIP, although the observed effect was relatively small.

## Data availability statement

The datasets presented in this study can be found in online repositories. The names of the repository/repositories and accession number(s) can be found in the article/**Supplementary Material**.

## Author contributions

ARG, SS, AG, CL, and CS conceived the research. ARG and JG performed the experiments. ARG, FC, KH, H-JR, and CS analyzed the data. ARG, SS, AG, CS, FC, and CL evaluated and interpreted the data. All authors contributed to manuscript writing and reviewing. All authors contributed to the article and approved the submitted version.
