# Supplementary material for: Anaerobutyricum hallii promotes the functional depletion of a food carcinogen in diverse healthy fecal microbiota
Source: Front Microbiomes. 2023 Sep 18;2:1194516. doi: 10.3389/frmbi.2023.1194516 (PMC12993610; doi:10.3389/frmbi.2023.1194516)
Supplement: Supplementary file 2 [file DataSheet_2.docx]

**Table S1. PERMANOVA table evaluating the association between taxonomic structure or composition of the fecal microbiota and co-variable**s. Jsd: Jensen-Shannon Divergence, wjaccard: weighted Jaccard, bjaccard: binary Jaccard.

| Community  Distance | Variable | Df | Sum Of Sqs | R2 | F | p value |
| --- | --- | --- | --- | --- | --- | --- |
| jsd | Enterotype | 2 | 0.115 | 0.373 | 5.949 | 0.001 |
| jsd | Gender | 1 | 0.008 | 0.025 | 0.785 | 0.543 |
| jsd | age | 1 | 0.009 | 0.029 | 0.929 | 0.461 |
| jsd | diet | 1 | 0.024 | 0.076 | 2.428 | 0.061 |
| jsd | 6h PhIP transformation | 1 | 0.021 | 0.069 | 2.19 | 0.074 |
| jsd | 24h PhIP transformation | 1 | 0.015 | 0.049 | 1.571 | 0.169 |
| jsd | Residual | 12 | 0.116 | 0.376 | NA | NA |
| jsd | Total | 19 | 0.309 | 1 | NA | NA |
| wjaccard | Enterotype | 2 | 0.789 | 0.219 | 2.565 | 0.001 |
| wjaccard | Gender | 1 | 0.133 | 0.037 | 0.866 | 0.568 |
| wjaccard | age | 1 | 0.128 | 0.035 | 0.831 | 0.63 |
| wjaccard | diet | 1 | 0.219 | 0.061 | 1.422 | 0.108 |
| wjaccard | 6h PhIP transformation | 1 | 0.25 | 0.069 | 1.627 | 0.062 |
| wjaccard | 24h PhIP transformation | 1 | 0.198 | 0.055 | 1.287 | 0.173 |
| wjaccard | Residual | 12 | 1.845 | 0.512 | NA | NA |
| wjaccard | Total | 19 | 3.606 | 1 | NA | NA |
| bjaccard | Enterotype | 2 | 0.25 | 0.106 | 1.049 | 0.34 |
| bjaccard | Gender | 1 | 0.116 | 0.049 | 0.971 | 0.512 |
| bjaccard | age | 1 | 0.187 | 0.079 | 1.576 | 0.021 |
| bjaccard | diet | 1 | 0.12 | 0.051 | 1.008 | 0.443 |
| bjaccard | 6h PhIP transformation | 1 | 0.15 | 0.063 | 1.261 | 0.134 |
| bjaccard | 24h PhIP transformation | 1 | 0.125 | 0.053 | 1.053 | 0.35 |
| bjaccard | Residual | 12 | 1.428 | 0.604 | NA | NA |
| bjaccard | Total | 19 | 2.364 | 1 | NA | NA |

**Table S2.** Partial redundance analysis (db-RDA) table evaluating the association between microbiota structure (Jensen-Shannon Divergence) and PhIP transformation while controlling for differences in microbiota related to enterotype.

| terms | Df | Sum of Squares | F | Pr. F. |
| --- | --- | --- | --- | --- |
| Model | 2 | 0.15 | 1.017 | 0.447 |
| Residual | 15 | 1.104 |  |  |


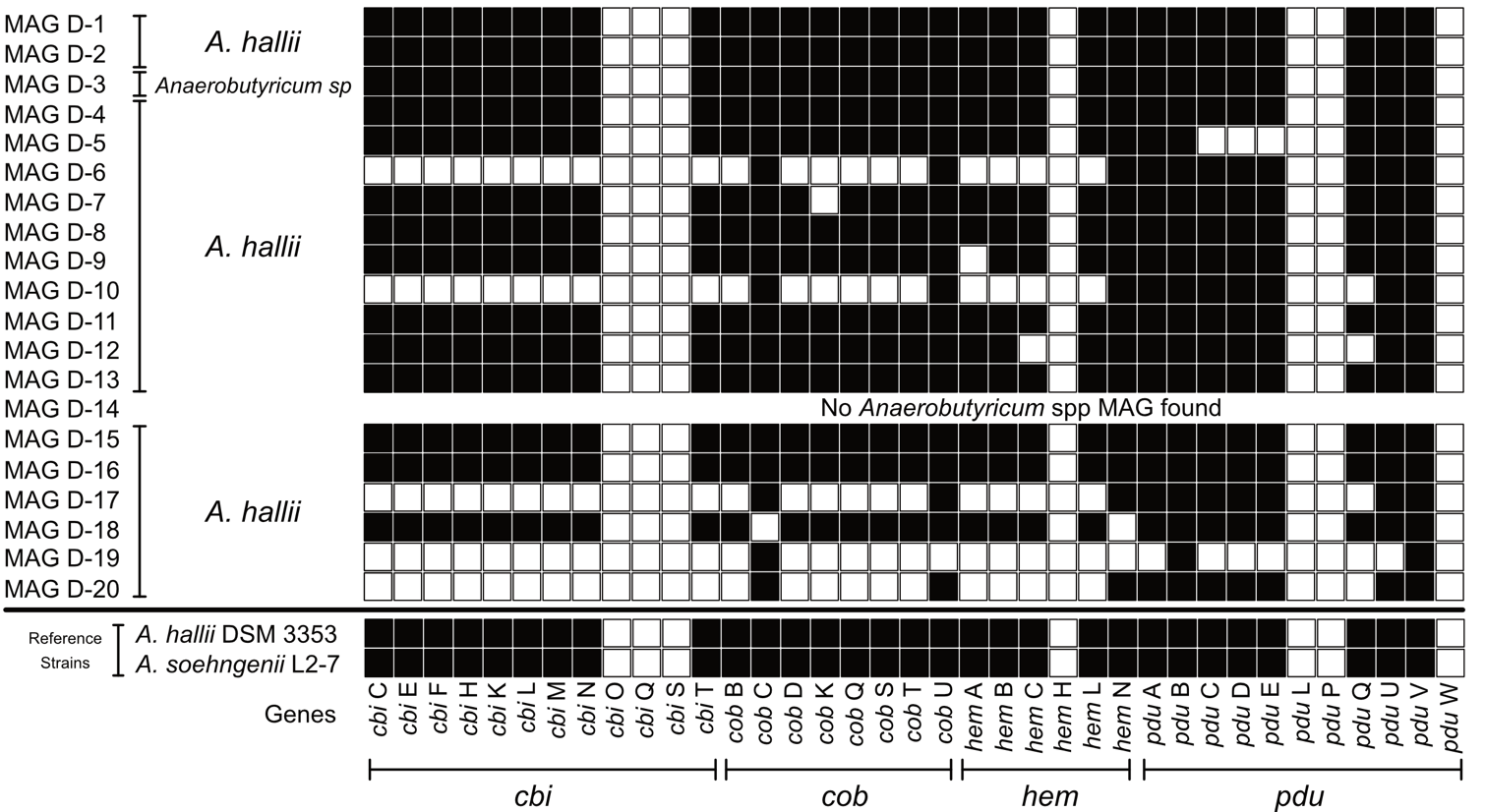


**Figure S1. Comparison of the *Anaerobutyricum* MAGs extracted from the fecal metagenomes of 20 healthy adult human donors.** Taxonomy assigned to each MAG and gene matrix showing presence (black) or absence (white) of the genes of the *pdu-cob-cbi-hem* operon required for acrolein production in each *Anaerobutyricum* MAG recovered from donor microbiota and the reference strains *A. hallii* DSM3353 and *A. soehngenii* L2-7.


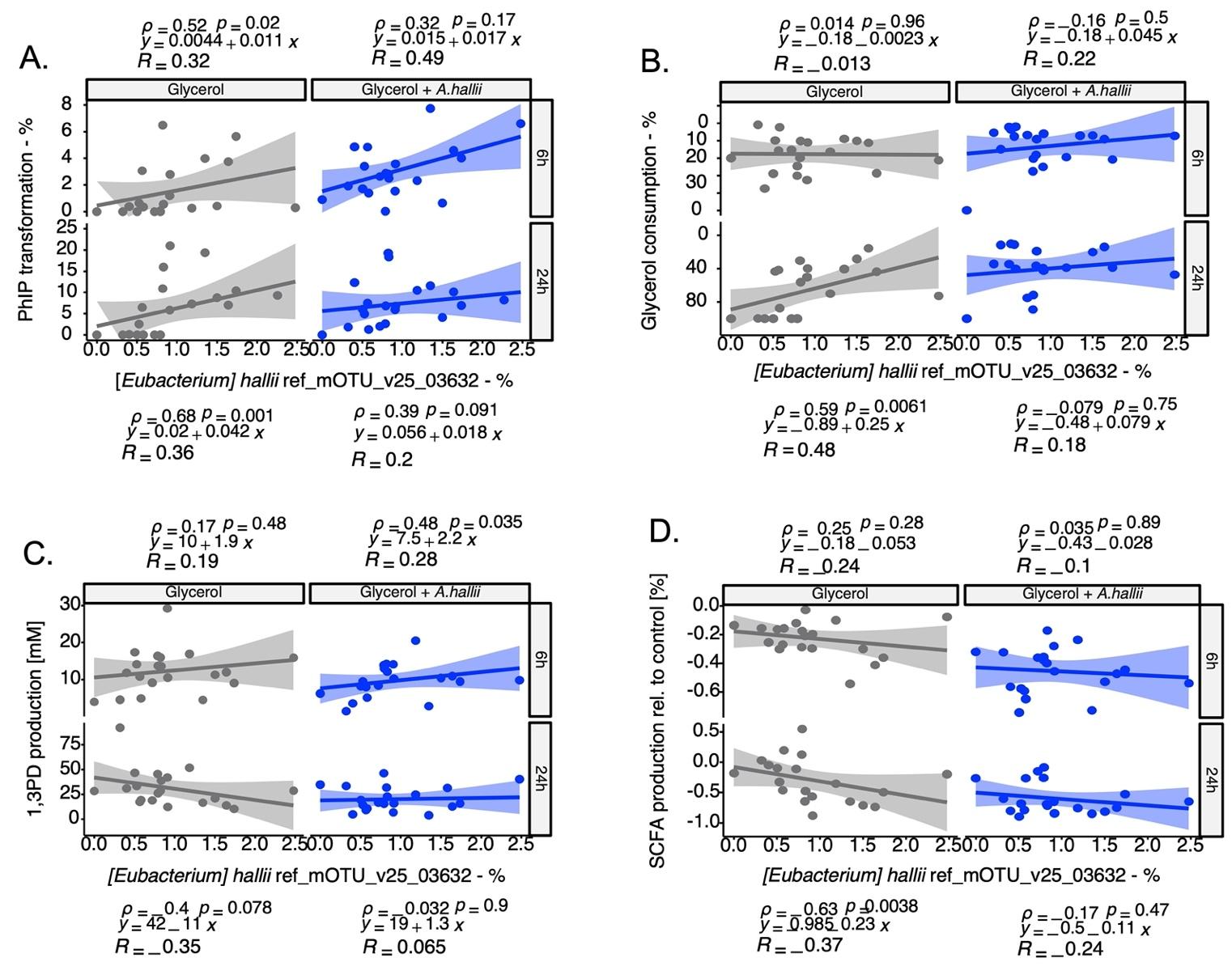


**Figure S2. Correlation between the resident *A. hallii* proportion in the fecal microbiota samples, and PhIP transformation (A), glycerol consumption (B), 1,3 PD production (C) and SCFA production relative to control (D).** Each fecal sample of 20 adult donors was grown in batch fermentations carried out in presence of 100mM glycerol (grey series) or glycerol + *A.hallii* (blues series) and data collected after 6 and 24 hours. For each plot, regression equation, Spearman’s ρ and p values as well as Pearson’s R are reported.

**
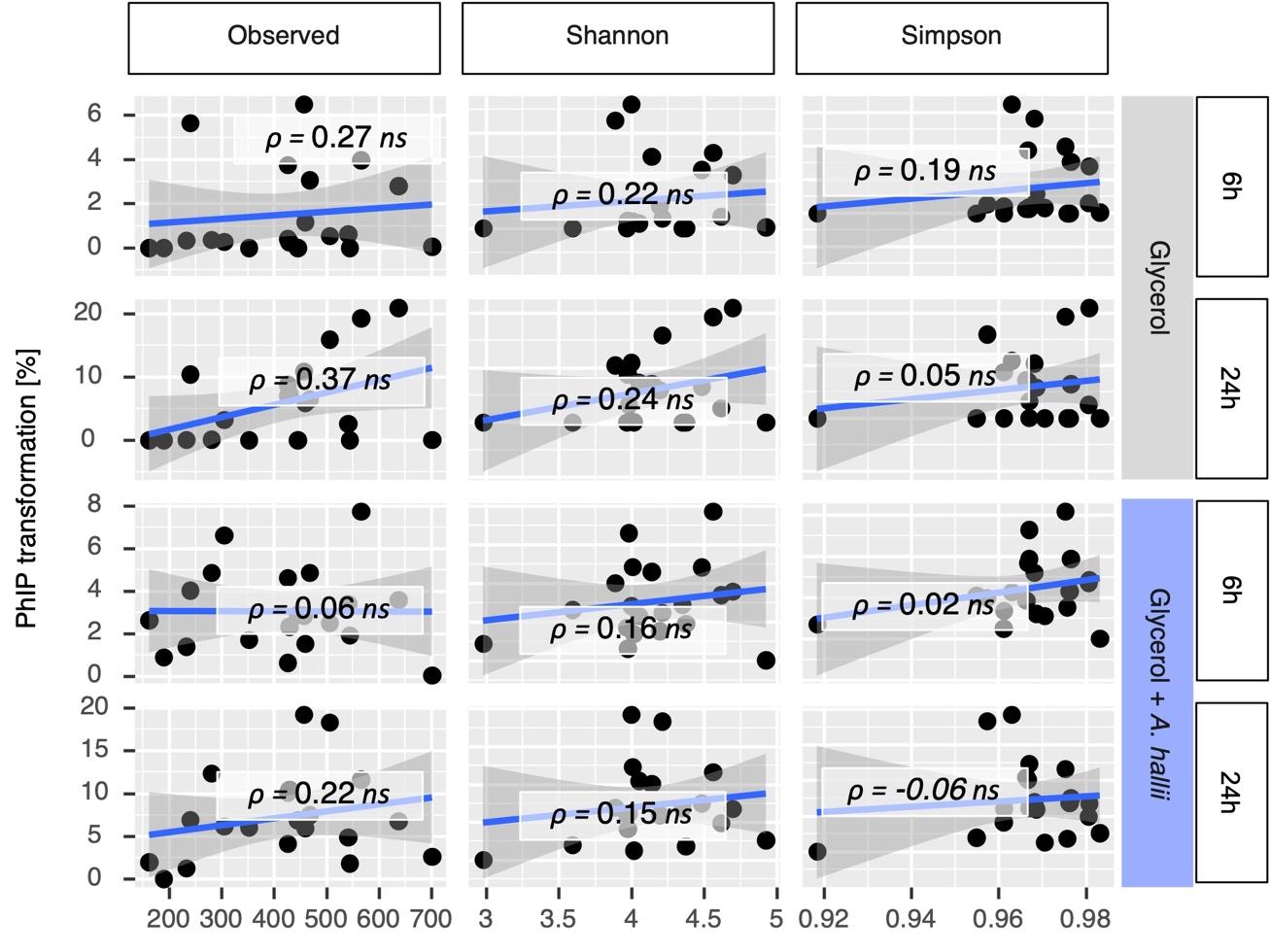
**

**Figure S3. Spearman-ranked correlations between alpha diversity indexed (Observed species, Shannon diversity and Simpson) and PhIP transformation in all tested conditions.**

**SUPPLEMENTARY METHODS**

**Composition of batch fermentation medium.**

Nutritive McFarlane medium adapted to batch fermentation conditions (Bircher *et al.*, 2018a) was used and its composition was as follows (in g L^-1^, all components obtained from Sigma-Aldrich, Buchs, Switzerland, unless stated otherwise): cellobiose (1.0), arabinogalactan from larch wood (1.0), potato starch (1.9), amicase (3.0), meat extract (1.5), yeast extract (4.5), porcine mucin (4.0), NaHCO_3_ (9.0), NaCl (4.5), KCl (4.5), hemin (0.05), MgSO_4_ (0.61), CaCl_2_∙2H_2_O (0.1), MnCl_2_∙4H_2_O (0.2), FeSO_4_∙7H_2_O (0.005), ZnSO_4_∙7H2O (0.1) , KH_2_PO_4_ (2.0) and KH_2_PO_4_ (3.0, VWR International. Dietikon, Switzerland), xylan from oat spelts (1.0, Chemie Brunschwig. Basel, Switzerland), inulin (0.5, RPN Food technologie. Sursee, Switzerland), Bacto tryptone (5.0, Becton Dickinson. Allschwil, Switzerland) and bile salt (0.4, Thermo Fisher Diagnostics. Pratteln, Switzerland). The medium was supplemented with 1 g cysteine-HCl and with a short-chain fatty acid (SCFA) mix for a final concentration of 33 mM acetate, 9 mM propionate, and 1 mM isovalerate, isobutyrate and valerate. A vitamin solution was also added for final concentrations (in µg L^-1^) of biotine (10), cobalamin (10), p-aminobenzoic acid (30), folic acid (50) and pyridoxamine (150, VWR International). The pH of the medium was adjusted to 7.2 using 5 M NaOH before boiling for 15 min and gassing with CO2 for 10 more min. Medium (20 mL) was transferred to 50 mL serum flasks (InfoChroma AG. Switzerland) under CO_2_ flow and closed with a rubber septum and an aluminum cap prior autoclaving.

**Preparation of *A. hallii* DSM3353 cultures**

Yeast extract – casein hydrolysate – fatty acids (YCFA) medium (Bircher *et al.*, 2018b) was used to grow *A. hallii*. The media composition contained (in g L^-1^, all components obtained from Sigma-Aldrich. Buchs, Switzerland, unless stated otherwise): amicase (10.0), NaHCO_3_ (4.0), hemin (0.01), NaCl (0.09), MgSO_4_ (0.09), CaCl_2_ (0.09), K_2_HPO_4_ (0.45), (NH_4_)_2_SO_4_ (0.09), KH_2_PO_4_ (0.45)_,_ yeast extract (2.5, VWR International), and 1 µg L^-1^ resazurin. The medium was supplemented with 50 mM glucose and the same SCFA mix and vitamin solution as for McFarlane medium. Medium (10 mL) was filled in 15 mL Hungate tubes (Milian. Vernier, Switzerland) and closed with a rubber septum and a plastic threaded cap. Cryopreserved cultures of *A. hallii* DSM 3353 (Bircher *et al.*, 2018a) were reactivated in an anaerobic chamber (10% CO_2_, 5% H_2_ and 85% N_2_) (Coy Laboratories. Grass Lake, MI, USA) by resuspension in 1 mL of YCFA media, and were transferred to 10 mL YCFA media in Hungate tubes for incubation at 37 °C.

**Additional microbiota community analysis**. Genus level relative abundances were used to compute Jensen-Shannon divergence (phyloseq::distance("jsd")) and subjected to Partitioning Around Medoids (PAM) clustering algorithm. The optimal number of clusters was determined as previously described (CH, fpc::pamk((genus_JSD_dist), krange = 2:6, criterion = "ch").

**SUPPLEMENTARY REFERENCES**

Bircher, L., Schwab, C., Geirnaert, A., and Lacroix, C. (2018a) Cryopreservation of artificial gut microbiota produced with in vitro fermentation technology. *Microbial biotechnology* **11**: 163-175.

Bircher, L., Geirnaert, A., Hammes, F., Lacroix, C., and Schwab, C. (2018b) Effect of cryopreservation and lyophilization on viability and growth of strict anaerobic human gut microbes. *Microbial biotechnology* **11**: 721-733
